# Research progress of the CXCR4 mechanism in Alzheimer's disease

**DOI:** 10.1002/ibra.12026

**Published:** 2022-03-03

**Authors:** Qiu‐Lin Wang, Chang‐Le Fang, Xue‐Yan Huang, Lu‐Lu Xue

**Affiliations:** ^1^ Department of Clinical Medicine Chongqing Medical University Chongqing China; ^2^ Department of Anesthesiology Southwest Medical University Luzhou Sichuan China; ^3^ Department of Anesthesiology The Affiliated Hospital of Zunyi Medical University Zunyi Guizhou China; ^4^ State Key Laboratory of Biotherapy of Sichuan University Chengdu Sichuan China

**Keywords:** Alzheimer's disease, astrocytes, CXCL12, CXCR4, microglia

## Abstract

Alzheimer's disease (AD) is a degenerative brain disease with complex clinical manifestations and pathogeneses such as abnormal deposition of beta‐amyloid protein and inflammation caused by the excessive activation of microglia. CXC motif chemokine receptor type 4 (CXCR4) is a type of G protein‐coupled receptor that binds to CXC motif ligand 12 (CXCL12) to activate downstream signaling pathways, such as the Janus kinase/signal transducer and activator of transcription and the renin–angiotensin system (Ras)/RAF proto‐oncogene serine (Raf)/mitogen‐activated protein kinase/extracellular‐regulated protein kinase; most of these signaling pathways are involved in inflammatory responses. CXCR4 is highly expressed in the microglia and astrocytes; this might be one of the important causes of inflammation caused by microglia and astrocytes. In this review, we summarize the mechanism and therapeutics of AD, the structures of CXCR4 and the CXCL12 ligand, and the mechanisms of CXCR4/CXCL12 that are involved in the occurrence and development of AD. The possible treatment of AD through microglia and astrocytes is also discussed, with the aim of providing a new method for the treatment of AD.

## Graphical abstract

- We summarize the mechanism of Alzheimer's disease (AD).
- We summarize the mechanism of CXC motif chemokine receptor type 4 (CXCR4)/CXC motif ligand 12 (CXCL12) and the structures of ligands CXCR4 and CXCL12, which are involved in the occurrence and development of AD.
- The possible treatment of AD through microglia and astrocytes is also described, with the aim of providing necessary evidence for the subsequent treatment of AD.

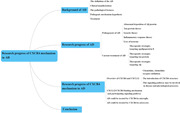

## 1 INTRODUCTION OF ALZHEIMER'S DISEASE

Alzheimer's disease (AD) is a degenerative brain disease and it has long been one of the great toughness of medicine. The duration of disease varies, and the prevalence of AD gradually increases with age, which is the most common cause of dementia.^1^ It is clinically characterized by cognitive dysfunction, psychiatric symptoms, and behavioral disorders, and even a gradual decline in the ability to carry out daily living activities. The Delphi study estimated that currently, worldwide, 24.3 million people have dementia, with 4.6 million new cases of dementia diagnosed every year (one new case every 7 s). It is predicted that the number of AD patients will reach 81 million by 2040.^2^ The main pathological features of AD include deposition of β amyloid (Aβ) plaques and nerve fiber tangles.^3^ Considered as a heterogeneous disease, the disease may be correlated with family history, head trauma, low educational level, thyroid disease, aging, viral infection, and so forth. There are many hypotheses in relation to the pathogenesis of AD, such as abnormal deposition of Aβ, tau protein theory, genetic theory, and so forth, but none of them have been fully confirmed.^4^ At present, a variety of treatments are available for AD, such as granulocyte colony‐stimulating factor and CXCR4 antagonist, mobilization of hematopoietic stem cells to peripheral blood, β secretase inhibitors and other drugs to reduce Aβ load, neurotrophic factors, and immunotherapy, but so far, there is no effective treatment for AD.^5^ Therefore, we summarized the pathogenesis and treatment of AD, as well as the potential treatment methods through CXCR4 to provide necessary evidence for the subsequent treatment of AD.

## 2 RESEARCH PROGRESS OF AD

### 2.1 Pathogenesis of AD

#### 2.1.1 Abnormal deposition of Aβ protein

Amyloid precursor protein (APP) is a complete protein located in the plasma membrane. Relevant studies have shown that APP plays an important role in regulating the survival, movement, and growth of cells, as well as the growth and function of neurites.^6^ APP could be cut by β‐secretase and γ‐secretase and produce insoluble Aβ fibrils to decompose the Aβ clumps or plaques in the synaptic cleft, which could destroy the synapses and interfere with the synaptic signal. Generally, Aβ are deposited in the basal, temporal, and orbitofrontal neocortex regions of the brain, spreading to the hippocampus and cerebral cortex at critical stages.^7^ Aβ induces overactivation of kinases, which include a key kinase that plays a role in the overphosphorylation of tau protein, overactivation of microglia, and local inflammatory responses.^8^, ^9^ Also, Aβ protein is toxic to surrounding synapses and neurons, and this could cause nerve cell death and abnormal deposition of plaques. It also could disrupt the sleep–wake cycle and usually precedes the presence of amyloid plaques. The duration of rapid eye movement (REM) sleep episodes is reduced, resulting in cumulative REM sleep deprivation. Patients suffer from insomnia at night and then increased daytime sleep, resulting in the disappearance of day–night changes.^10^

#### 2.1.2 Tau protein deposition hypothesis

AD is also characterized by the presence of nerve fiber tangles, which are the result of microtubule‐associated tau protein hyperphosphorylation.^11^ The microtubule‐binding domain on tau protein can bond with tubulin to form mature and stable microtubules and a bridge network between adjacent microtubules. When excessive Aβ plaques are deposited in the brain, tau proteins come into contact with the released kinases, resulting in hyperphosphorylation and Aβ oligomerization.^12^ In addition, due to the hyperphosphorylation of tau protein, the stability of microtubules decreases, and the subunits of tubules decompose and separate from the cytoskeleton to form a double‐helix structure. As a result, the cytoskeleton structure is decomposed and destroyed, and then gather to form tangles. Highly insoluble plaques are located in the cytoplasm and neurons, which can lead to decreased signal transmission between neurons and neuronal apoptosis.^13^ Tau hyperphosphorylation is regulated by a variety of kinases in addition to Aβ in the brain, such as glycogen synthase kinase 3, extracellular Aβ‐activated cyclin‐dependent kinase 5, protein kinase C (PKC), and protein kinase A (PKA).^14^

#### 2.1.3 Genetic hypothesis

Apolipoprotein E (APOE) is a key enzyme responsible for the production of Aβ plaque, which is involved in the regulation of Aβ production and affects the clearance of Aβ in astrocytes and neurons, leading to the deposition of Aβ. *APOE*, composed of *PSEN1, PSEN2, Aph1*, and *Nicastrin*, is a key gene encoding the APOE enzyme. Psen is an aspartic protease and serves as the catalytic core of the enzyme. *PSEN1* and *PSEN2* are key genes encoding Psen. In AD patients, *PSEN1* and *PSEN2* are prone to mutation, leading to the generation of more toxic amyloid.^15^ Triggering receptor expressed on myeloid cells‐2 (TREM2) is a transmembrane glycoprotein composed of extracellular immunoglobulin‐like domains, transmembrane domains, and a cytoplasmic tail, encoded by *TREM*. TREM2 is mainly involved in the regulation of two signaling pathways, one of which is the regulation of phagocytosis signals and the cell phenotype regulation of reactive glial cells, which can bind anionic carbohydrates, bacterial products, and phospholipids. Furthermore, intracellular signals are transmitted through the related transmembrane adaptor DAP1255 and lead to further phosphorylation of downstream mediators, which is mainly related to the clearance of cell debris and Aβ in AD. Another mechanism is mainly related to the suppression of inflammatory response, mainly involving the suppression of cytokine secretion.^16^ Guerreiro and Hardy^17^ found that rare variants of TREM (P.r47h) are significantly correlated with the risk of development of AD in a study carried out on patients with AD. It can be reasonably surmised that the *TREM* gene mutation leading to the reduction of TREM2 is the key factor responsible for the occurrence and development of AD.

#### 2.1.4 Inflammatory response hypothesis

Microglia are phagocytes in the central nervous system (CNS), mainly responsible for maintaining neuronal plasticity and synaptic remodeling, clearing Aβ, and generating inflammatory responses.^18^ Microglia are the smallest glia cells in CNS, and their cell body is spindle or oval shaped with a spinous process on the surface.^19^ Microglia are the main effectors in the inflammatory process of the CNS. When there are foreign antigen substances or neuronal cellular debris, microglia can quickly be activated and produce different kinds of cytokines to promote the occurrence and development of an inflammatory reaction, activate T cells through antigen presentation, leading to nervous system damage, and induce the aggregation of resting microglia.^20^, ^21^ Abnormal deposition of Aβ and hyperphosphorylation of tau lead to synaptic damage and increased reactive oxygen species stress. Cell surface receptors of microglia, such as Toll‐like receptor (TLR), CD36, CD14, α6β1 integrin, CD47, and CXCR4, can bind to Aβ. Activated microglia can engulf Aβ and nerve fiber tangles and secrete a variety of proinflammatory cytokines and chemokines through the Janus kinase/signal transducer and activator of transcription (JAK/STAT) signaling pathway, such as nitric oxide, tumor necrosis factor‐α, and interleukin‐6 (IL‐6), leading to the inflammatory response of AD.^22^ Inflammatory response is influenced by a variety of factors, such as local cytokine concentrations and mutations in TREM. The TREM mutation can change the phenotype of microglia, resulting in decreased phagocytosis of microglia to Aβ in AD, leading to the accumulation of Aβ and development of AD.^23^

#### 2.1.5 Loss of neurons

**Figure 1:**
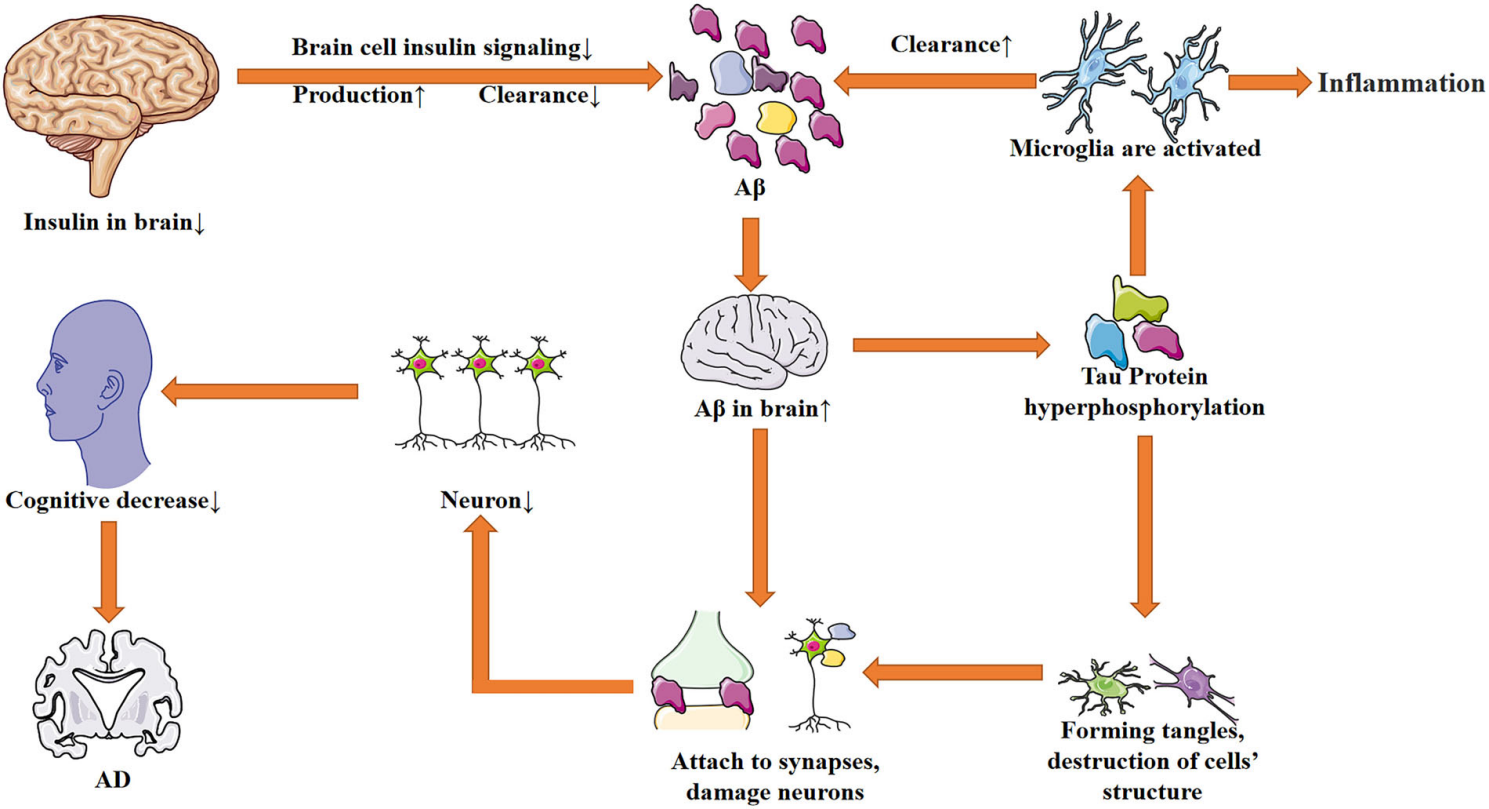
The pathogenesis of AD. Because of increasing insulin levels in the brain, the insulin signaling pathway is blocked, and then the production of Aβ is increased and the clearance of Aβ is decreased, leading to the accumulation of Aβ in the brain. Aβ can damage synapses, lead to signal transmission obstruction, damage neurons, and activate microglia, which can produce inflammation. A decrease in the number of neurons leads to a decline in cognitive function. Activated microglia can phagocytize Aβ. Aβ, β amyloid AD, Alzheimer's disease [Color figure can be viewed at wileyonlinelibrary.com]

Neuronal loss is a basic feature of the pathogenesis of AD. The loss process begins in the preclinical stage, before the appearance of clinical neuropathological features. In the early stage, the number of neurons in various brain regions decreases to varying extents and in the late stage. The whole brain might be involved in the stage, especially loss of neurons in the hippocampal CA1 region.^24^ The main types of missed neurons are pyramidal neurons, interneurons, and cholinergic neurons. The loss of pyramidal neurons is related to intracellular Aβ and tau protein.^25^ The overexpression of APP is a major cause of the decrease of pyramidal neurons. The APP23 model expressing human APP under the control of the thy‐1 promoter in mice showed evidence of the decrease of neurons. Related studies have demonstrated that the pyramidal neurons in AD patients were reduced, and the number of amyloid and pyramidal neurons was negatively correlated with it.^26^ Tau also causes loss of neurons. Accelerated plaque formation, neurofibrillary degeneration, and neuron loss were found in Tg2576 mice after hybridization expressing human 4‐repeat tau.^27^ Early loss of cholinergic neurons in the basal forebrain leads to reduced cholinergic transmission, which is an important cause of cognitive decline in AD patients. German et al.^28^ conducted studies in a variety of genetic AD mouse models. The results showed that the decreased density of cholinergic nerve endings at 4 months of age in homozygous patients was one of the causes of cognitive decline in AD patients (the above pathogenesis is shown in Figure 1).

### 2.2 Current treatment of AD

#### 2.2.1 Therapeutic strategies targeting APOE

The main role of the APOE subtype is to control brain lipid transport, neuronal signaling, mitochondrial function, glucose metabolism, and neuroinflammation. It is now believed that the onset of AD neurodegeneration occurs when apolipoprotein binds to various cell surface receptors to transport lipids and lipophilic Aβ peptides. APOE plays an important role in the production, aggregation, clearance of Aβ, tau‐mediated neurodegeneration, neuroinflammation, α‐synaptic nucleolysis, lipid metabolism, and synaptic plasticity.^29^ The therapeutic strategy targeting APOE was mainly to reduce the harmful effects of APOE4 and rebuild the protective capacity of APOE. There were studies suggesting that increasing APOE lipidation might be the key to apolipoprotein‐based therapy, rather than focusing on increasing APOE. APOE lipidation is mediated by the ATP binding box transporter A1 (ABCA1).^30^ Wahrle et al.^31^ used a PDAPP transgenic AD mouse model and found that the ABCA1 deletion led to increased Aβ peptide deposition and overexpression of ABCA1 inhibited Aβ deposition. It could be concluded that increased APOE lipidation reduces the Aβ load. APOE could lead to the deposition of Aβ and there was a corollary for the treatment of APOE: reducing the level of APOE was conducive to reducing the deposition of amyloid.^32^ In addition, some studies have found that anti‐APOE immunotherapy could also inhibit Aβ accumulation.^33^

#### 2.2.2 Therapeutic strategies targeting tau protein

The expression of Tau protein and the process of post‐translational modification, aggregation and clearance provide opportunities for the treatment of AD. Inhibition of tau protein expression represents a good method to treat AD. Small interfering RNA antisense oligonucleotides can reduce the expression of tau protein. In cell and animal models, siRNA has been found to reduce tau pathology and damage.^12^ The main approach of anti‐tau therapy involves inhibiting the aggregation of kinases or tau proteins or stabilization of microtubules. For example, methylene blue can prevent the aggregation of tau monomer by capturing the nonaggregation conformation of tau monomer in vitro.^34^ However, at present, most tau‐targeted therapies involve immunotherapy, which has good therapeutic potential. Targeted active immunity to single or multiple phosphoric acid epitopes and amino‐terminal has been shown to reduce tau pathologic processes.^35^ However, inducing antibodies to natural proteins carries the risk of an adverse immune response. Studies indicated that the tau vaccine could be toxic when used in combination with a strong helper T adjuvant. Milder adjuvants could be used to reduce the incidence of this adverse reaction.^36^

#### 2.2.3 Therapeutic strategies targeting Aβ

The abnormal deposition of Aβ was one of the pathogeneses of AD, and the study of the cause and process of the deposition undoubtedly represents a mode of treatment of AD. Aβ is a protein hydrolyzed by APP under the action of secretase, which inhibits the production process of Aβ, and the aggregation process could interfere with the pathology of AD.^37^ However, due to the wide range of secretase substrates, secretase could enter the blood–brain barrier, which leads to obstacles to the clinical development of such drugs. Promoting Aβ clearance was also a novel therapeutic approach. Active and passive immunizations have been developed to facilitate the Aβ removal process. The immune‐mediated mechanisms that promote the removal of Aβ might be the dissolution of Aβ by antibody binding and promotion of phagocytosis of microglia on opsonin Aβ.^38^ Active immunotherapy is less costly, but it is difficult to control antibody concentrations during treatment. Passive immunotherapy can quickly control antibody concentrations (Table 1).

**Table 1:** The treatment of AD

Reference	IF	Object	Intervening measures	Results
Barry Reisberg et al. (2003)	70.67	AD	Patients with moderate‐to‐severe Alzheimer's disease were randomly assigned to receive placebo or memantine	Antiglutamatergic treatment reduced deterioration in AD
Serge Gauthier et al. (2016)	59.102	AD	Patients were randomly assigned to the experimental group and the control group to receive different doses of LMTM (a selective inhibitor of tau protein aggregation)	Gastrointestinal and urinary effects were the most common adverse events with both high doses of LMTM
Mark H Tuszynski et al. (2015)	12.321	AD	NGF gene therapy with in vitro or in vivo gene transfer in AD patients	Degenerating neurons in the AD brain responded to NGF

*Note*: Research on the treatment of AD in recent years.

Abbreviations: AD, Alzheimer's disease; LMTM, leuco‐methylthioninium bis; NDF, nerve growth factor.

## 3 RESEARCH PROGRESS OF THE CXCR4 MECHANISM IN AD

### 3.1 Overview of CXCR4 and CXCL12

Chemokine receptors are a class of G‐protein‐coupled receptors (GPCRs) with seven transmembrane domains. GPCR is the largest family of surface receptors and its basic composition includes an extracellular region, a through membrane (TM) region, and an intracellular region. The extracellular region is composed of an N‐terminal and three extracellular loops (ECL), the TM region has seven TMα helices, and the intracellular region contains three intracellular loops.^39^ GPCR is a multifunctional signaling molecule on the surface of cells, which is involved in communication between cells and the perception of the external world. It was not difficult to understand that GPCR was involved in the pathogenesis of many diseases.^7^ CXCR4 is an evolutionarily highly conserved member of the GPCR family expressed on peripheral blood monocytes, B cells, naive T cells, and so forth.^40^ The CXCR4 coding gene is located on human chromosome 2q21, encoding 35 amino acid residues and a highly conserved coding sequence. Its ligand CXCL12 is a homeostasis chemokine, which is expressed in the liver, spleen, pancreas, and heart. Chemokines are low‐molecular‐weight (8000–10,000) proteins belonging to the cytokine superfamily. They are involved in leukocyte transport in physiological immune surveillance and inflammatory cell recruitment in host defense. Chemokines are key regulators of cell migration in development, dynamic balance, and immune surveillance. The amine‐terminal domain of CXCL12 binds to the second ECL of CXCR4 and activates downstream signaling pathways, such as calcium flux activation, focal component activation, pyruvate kinase‐2, PKC, and so forth. Through these pathways, inflammatory response is regulated and involved in a series of physiological and pathological processes such as tumorigenesis and metastasis, vascular extravasation, embryonic hematopoiesis, organogenesis, angiogenesis, and other embryonic growth and development, as well as immune monitoring.^41^, ^42^

### 3.2 CXCL12/CXCR4 binding mechanism and participating signaling pathway

**Figure 2:**
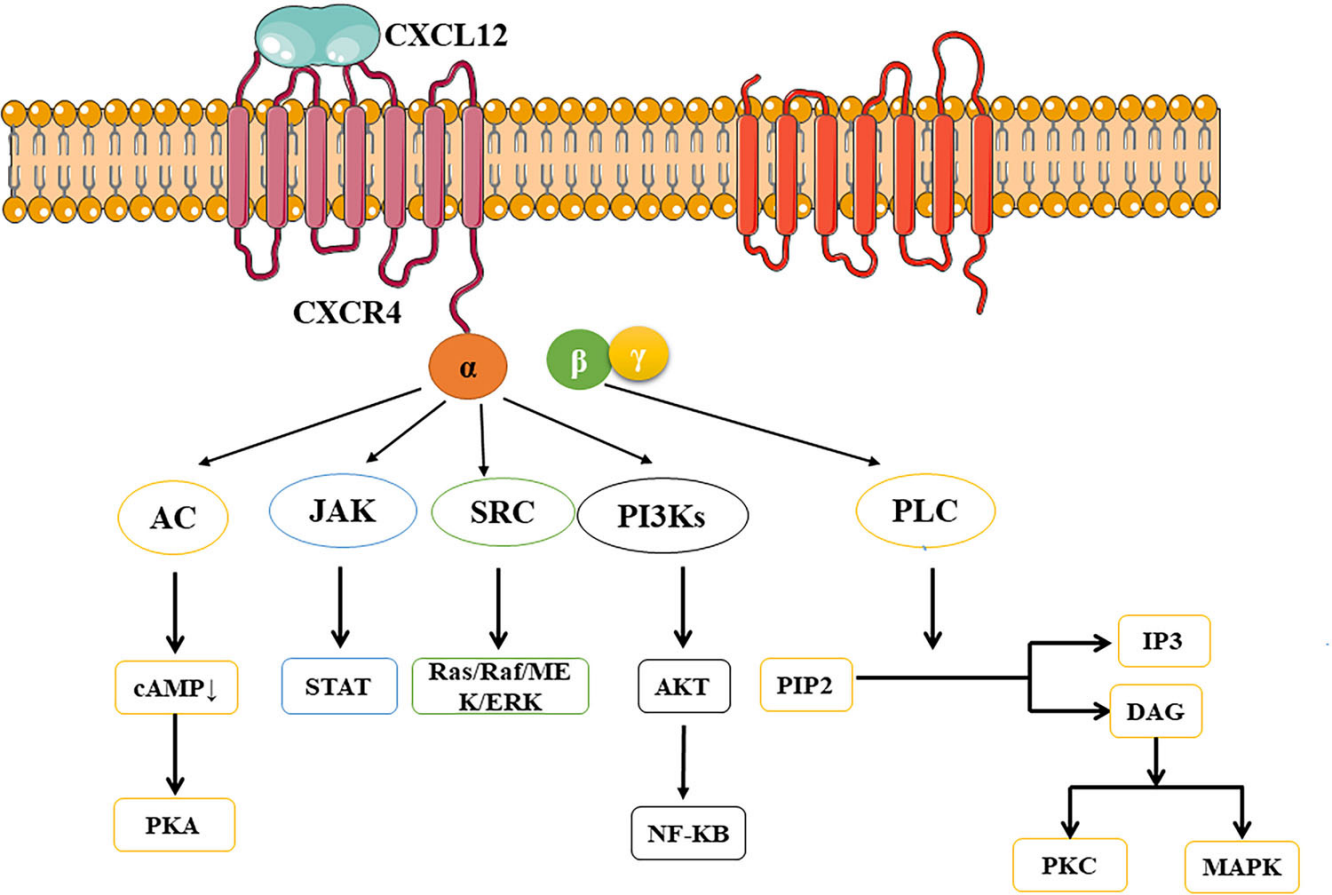
CXCL12/CXCR4 participating signaling pathways. AC, adenylate cyclase; cAMP, cyclic adenosine monophosphate; CXCL12, CXC motif ligand 12; CXCR4, chemokine receptor type 4; DAG, diglycerol; IP3, inositol 1, 4, 5‐triphosphate; JAK, Janus kinase; MAPK, mitogen‐associated protein kinase; NF‐κB, nuclear factor kappa B; PI3K, phosphatidylinosine 3 kinase; PKA, protein kinase A; PKC, protein kinase C; PIP2, phosphatidylinositol 4, 5‐diphosphate; STAT, signal transducer and activator of transcription [Color figure can be viewed at wileyonlinelibrary.com]

The binding of CXCL12 to CXCR4 occurs mainly through two mechanisms; the first step is the stable coupling of the corresponding receptor conformational change induced in the extracellular region, and the second step is the intracellular Gα subtype dissociation from the Gβ/Gγ dimer and the activation of the G protein trimer. Further conformational changes of the corresponding receptor were induced by the G protein trimer to form a stable CXCR4–CXCL12 couplet. Once activated, Gα I inhibits the production of adenylate cyclase and cyclic adenosine monophosphate (cAMP), and then inhibits PKA, stimulates the activity of Src family tyrosine kinases, activates the Ras/RAF/Raf/MEK/ERK pathway, and regulates the cell cycle by phosphorylation of adaptor protein (Src homology 2 domain‐containing) transforming protein (Shc). Meanwhile, phosphatidylinosine 3 kinases mediate CXCR4‐directed migration, which are activated by Gβγ and Gα subunits and regulate gene transcription, cell migration, and cell adhesion by phosphorylating protein kinase B α (AKT) to produce nuclear factor‐kappa B (NF‐κB).^43^, ^44^ In addition, the Gβ/Gγ dimer could trigger the activation of phospholipase C and catalyze the hydrolysis of phosphatidylinositol 4, 5‐diphosphate to inositol 1, 4, 5‐triphosphate and diglycerol (DAG). DAG promotes the activation of PKC‐ and mitogen‐associated protein kinase (MAPK).^45^ CXCR4 is sensitive to lipopolysaccharide (LPS) stimulation and has been shown to modulate the TLR4 signaling pathway. After LPS activation of TLR4, TLR4‐MyD88‐mediated signaling causes MAPK activation, which ultimately promotes NF‐κB signaling and the production of inflammatory mediators and cytokines. CXCR4 can regulate this process, and CXCR4 is sensitive to LPS stimulation, which can activate the NF‐κB pathway and produce inflammatory response.^46^ CXCL12/CXCR4 is widely expressed in peripheral and central nerves and plays an important role in regulating various processes of nervous system development, synaptic plasticity, and glial interaction. In brain disease, the expression of CXCL12/CXCR4 is upregulated in neurons, astrocytes, microglia, macrophages, and white blood cells, which may be involved in the occurrence and development of some neuropathy. Studies have shown that the CXCL12‐stimulated CXCR4 pathway regulates the activation of AKT, cAMP‐response element‐binding phosphorylation, and P53 levels and affects the process of aging and AD, suggesting that CXCR4 might be a new target and biomarker for the treatment of AD, but the specific mechanism remains to be further investigated^42^, ^47^ (CXCL12/CXCR4 participating signaling pathways are shown in Figure 2).

### 3.3 The role of CXCR4 mediation in microglia for AD

Microglia cells are phagocytes in the CNS. It has both proinflammatory and anti‐inflammatory effects on AD. The proinflammatory function mainly promotes neurotoxicity and accumulation of Aβ and the anti‐inflammatory function mainly promotes Aβ removal and neuroprotective function. Rangaraju and Dammer used flow cytometry and found that proinflammatory microglia appeared early in an AD mouse model, which were characterized by the expression of proinflammatory genes such as prostaglandin‐endoperoxide synthase 2, surface markers CD44 and potassium channel Kv1.3, and regulators NF‐κB and Stat1, while the anti‐inflammatory microglia expressed phagocytic genes somatomedin C (Igf1) and APOE and the surface markers of different regulators such as liver X receptor α/β (LXRα/β) on CXCR4.^48^ LXRα/β activator can block k+ channels and promote the nelproduction of anti‐inflammatory microglia, inhibit the production of proinflammatory microglia and enhance the phagocytosis of Aβ in the AD model. CHI3L1 (GP39 YKL40) is a glycoprotein belonging to the chitinase family and is secreted by a variety of cells, such as macrophages, dendritic cells, osteoclasts, and cells with high proliferative activity. It is linked to inflammation. Studies have shown that CHI3L1 is abundant in astrocytes, microglia, and infiltrating macrophages; CXCR4 level showed a positive correlation with CHI3L1.^49^ This further indicated that CXCR4 was related to the proinflammatory effect of microglia, but the specific mechanism of the association between CXCR4 and CHI3L1 needs to be investigated.

Relevant studies have shown that the JAK/STAT pathway activated by the CXCL12/CXCR4 axis promotes the inflammatory response of microglia in AD. Therefore, we could inhibit the inflammatory response of microglia by blocking this pathway.^50^ Under LPS stimulation, CXCR4 on microglia can activate the NF‐κB pathway, where NF‐κB is a key factor in upregulating the expression of proinflammatory genes and can inhibit the inflammatory response by inhibiting the activation of the NF‐κB pathway.^46^ However, there was another argument at the same time. In AD patients, the increased CXCR4 level and the decreased CXCL12 level are negatively correlated, and this relationship is particularly obvious in the hippocampus. CXCR4 might have a new ligand to bind to it and establish a new signaling pathway, leading to cognitive impairment and neuroinflammation. Macrophage migration inhibitory factor (MIF) might be a new ligand. The increased concentration of MIF was detected in the cerebrospinal fluid of an AD mouse model. We could inhibit the production of downstream signals by inhibiting the production and binding of new ligands to treat AD inflammation.^51^ We might be able to restore the CXCL12/CXCR4 axis by increasing CXCL12 levels in the brain, transforming the microglia response from proinflammatory into neuroprotective.^52^

**Figure 3:**
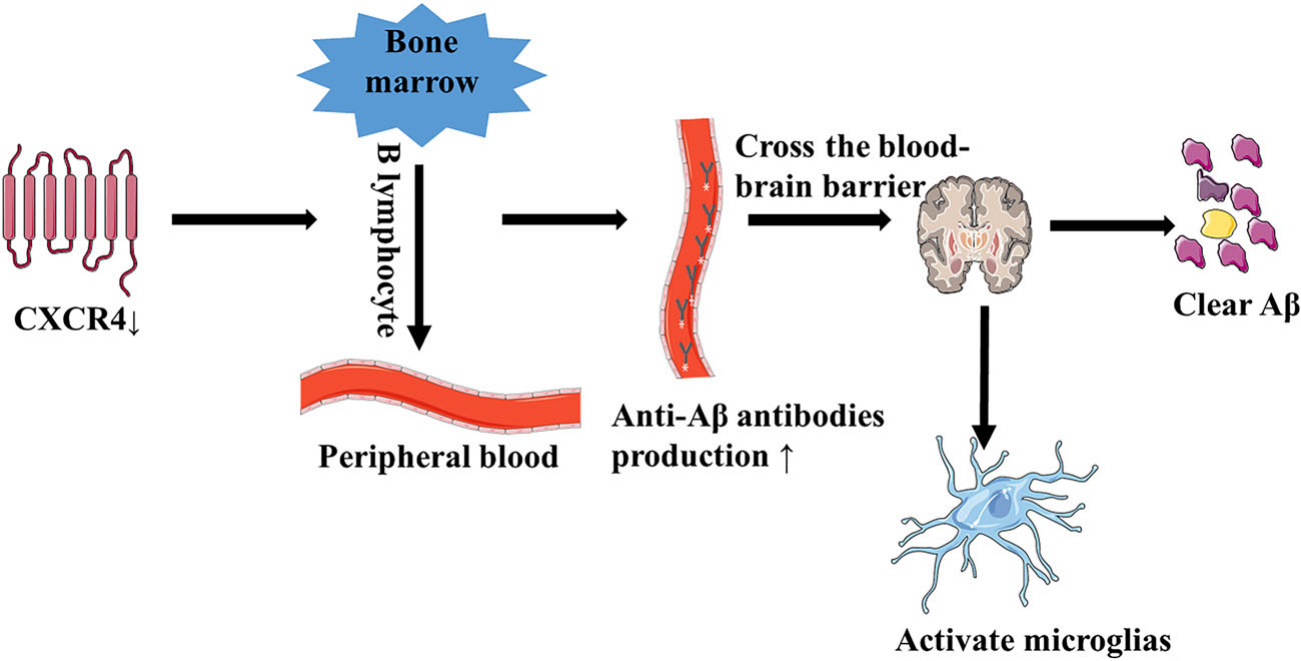
B‐cell migration. Decrease of CXCR4 caused the migration of B cells from the bone marrow to peripheral blood and an increase of anti‐Aβ antibody, which crossed the blood–brain barrier and entered the cerebrospinal fluid. Antibodies entering cerebrospinal fluid can clear Aβ and activate microglia, further increasing the clearance of Aβ. Aβ, β amyloid; CXCR4, chemokine receptor type 4 [Color figure can be viewed at wileyonlinelibrary.com]

The basic pathology of AD is an abnormal accumulation of Aβ and hyperphosphorylation of tau protein and its treatment depends on the clearance of Aβ. The activated microglia and the anti‐A β antibody have the effect of removing Aβ. In addition, the anti‐Aβ antibody also activates microglia. Anti‐Aβ antibodies are secreted by B cells. CXCR4 is a surface receptor for B cells, and the migration of immature B cells from the bone marrow to the peripheral blood is dependent on the downregulation of CXCR4. However, the upregulation of CXCR4 in patients with AD leads to a decrease in the migration of B cells and the secretion of anti‐A β antibodies, thereby increasing the accumulation of Aβ. This suggests a promising method for the treatment of AD: AD can be treated by reducing the expression of CXCR4 and increasing the migration of B cells. However, the mechanism by which antibodies from peripheral blood penetrate the blood–brain barrier into cerebrospinal fluid and how anti‐Aβ antibodies activate microglia remain to be studied.^53^, ^54^ Hematopoietic stem cells migrate from the bone marrow to peripheral blood, differentiate into microglia‐like cells, express microglia markers, and participate in Aβ phagocytosis. Kuroda et al.^55^ found that granulocyte colony‐stimulating factor and CXCR4 antagonists promoted the migration of hematopoietic stem cells to peripheral blood, differentiated into microglia, and participated in phagocytosis of Aβ, reducing Aβ burden. However, there are still some obstacles to the advancement of this treatment method to clinic and the mechanism by which migration can be promoted remains to be studied (B‐cell migration is shown in Figure 3. Research on CXCR4 and CXCL12 carried out over the recent years is shown in Table 2. Studies on microglia and AD over the recent years are shown in Table 3).

**Table 2:** CXCR4 and CXCL12 in AD

Reference	IF	Object	Intervening measures	Results
Thomas C. Beck et al. (2014)	10.892	B cells	Interrupt the CXCR4 signal	The export of immature B cells from BM is dependent on double downregulation of CXCR4
Yuval Gavriel et al. (2020)	3.517	AD	Combined use of AMD3100 and l‐lactic acid in the treatment of AD	The combined treatment resulted in a significant improvement in cognitive/memory functions and attenuated neuroinflammation
Simona Capsoni et al. (2017)	11.814	AD	Mimic the parenchymal delivery of hNGFp used in clinical trials and intranasal injection of the same dose of hNGFp	2hNGFp acts on glial cells and regulates inflammatory proteins by regulating CXCL12

*Note*: Research on CXCR4 and CXCL12 over the recent years.

Abbreviations: AD, Alzheimer's disease; BM, bone marrow; CXCL12, CXC motif ligand 12; CXCR4, chemokine receptor type 4; hNGFp, human painless nerve growth factor.

**Table 3:** Microglia and AD

Reference	IF	Object	Intervening measures	Results
Shoutang Wang et al. (2020)	10.892	AD	Mouse AD models included the use of the anti‐human TREM2 agonist mab AL002c	Reduced filamentous plaques and neurite dystrophy, impacted behavior, and tempered microglial inflammatory response
Alexander Kellner et al. (2009)	9.496	AD	Aβ, phagocytes, resting and activated microglia, and microbleeds in the central nervous system of AD patients were quantified and scored	Autoantibodies directed against beta‐amyloid may contribute toward controlling plaque burden
Masamichi Yokokura et al. (2010)	7.182	AD	Nootropic drug‐naive AD patients underwent a series of positron emission tomography, a compared with those in the brain regions with reduced glucose metabolism	Aβ accumulation is not always the primary cause of microglial activation

*Note*: Study on microglia and AD over the recent years.

Abbreviations: Aβ, β amyloid; AD, Alzheimer's disease; TREM2, triggering receptor expressed on myeloid cells‐2.

### 3.4 Role of CXCR4 mediation in astrocytes for AD

Several studies have suggested that CXCR4 and related downstream signaling pathways on astrocyte surface may be potential therapeutic targets for AD. Astrocytes are immune cells that exist in the CNS. Their main functions are to clear synaptic neurotransmitters, such as glutamate or gamma‐aminobutyric acid (GABA), participate in the formation of the blood–brain barrier, maintain the balance of ions inside and outside the cell, and provide nutrition for nerve cells.^56^ Astrocytes can be activated by Aβ deposition, and then secrete a variety of chemokines to bind corresponding receptors, such as MCP‐1/CCR2, CXCL12/CXCR4, MIP‐1α/CCR5, IP‐10/CXCR3, and so forth. The combination of chemokines and their receptors plays an important role in the pathological process of AD, such as inflammatory response, the death of neurons, and so forth.^57^, ^58^ CXCR4 is widely expressed on astrocytes and can be activated by CXCL12. The binding of CXCL12 and CXCR4 can activate JAK/STAT, NF‐κB, ERK, and other pathways and lead to an increase in the intracellular Ca2+ concentration in astrocytes, and secretion of glutamate and GABA to interfere with synaptic information transmission. McP‐1, IL‐8, and IP‐10 can also be induced by the ERK signaling pathway.^59^, ^60^ These chemokines could act as chemical inducers to cause the migration of astrocytes and neurons to sites of neuroinflammation. For example, IP‐10 promotes astrocyte aggregation around Aβ.^61^ Chemokines can also induce monocytes and migration of T cells of peripheral blood from the blood to the brain, causing brain inflammation.^62^ This suggested that we can reduce the inflammatory response of AD by preventing an increase in the intracellular calcium ion concentration of astrocytes or thereby preventing the secretion of substances such as glutamate, or by preventing the induction of chemokines.

CXCL12/CXCR4 binding can regulate neuronal excitability, signal propagation in the glial network, and synaptic transmission by enhancing glutamate release in astrocytes.^63^, ^64^, ^65^, ^66^ Glutamate is the main excitatory transmitter in the CNS, which has many physiological functions such as aiding learning and memory. However, high extracellular glutamate levels may lead to hyperactivation of postsynaptic neurons, which in turn leads to neuronal death. Astrocytes can provide the necessary metabolic support for neighboring neurons and other cells, while protecting neighboring cells by taking in excess glutamate and K+ and releasing growth factors, mitogens, and other important chemical messengers. This suggested that decreasing the extracellular glutamate concentration by increasing glutamate uptake by protective astrocytes may be a potential treatment for AD. Blocking the CXCR4‐mediated JAK/STAT pathway on astrocytes may be a potential treatment for AD. It has been shown that inhibition of the JAK/STAT pathway in astrocytes can reduce Aβ deposition in AD. SOCS3, an endogenous JAK/STAT inhibitor, was injected into the astrocytes of APP/PS1 or 3xTg mice by an AV vector. The results showed that blocking the JAK/STAT signaling pathway reduced glial reactivity, reduced amyloid deposition, and improved synaptic function and plasticity. In contrast, JAK2 subtypes expressing structural activity aggravate pathological and cognitive deficits in AD.^67^ This study demonstrated that blocking the CXCR4‐mediated JAK/STAT signaling pathway in astrocytes can improve the pathology of AD to a certain extent, which provided a new therapeutic avenue for the treatment of AD. However, Guillemaud et al.^68^ found that inhibition of the astrocyte JAK/STAT pathway did not reduce pathological amyloid deposition in 3xTg mice. This suggested that the treatment of AD by blocking the CXCR4‐mediated JAK/STAT pathway on astrocyte surface needs further experimental verification, but it is undoubtedly a potential treatment for AD.

## 4 CONCLUSIONS AND PROSPECTS

**Figure 4:**
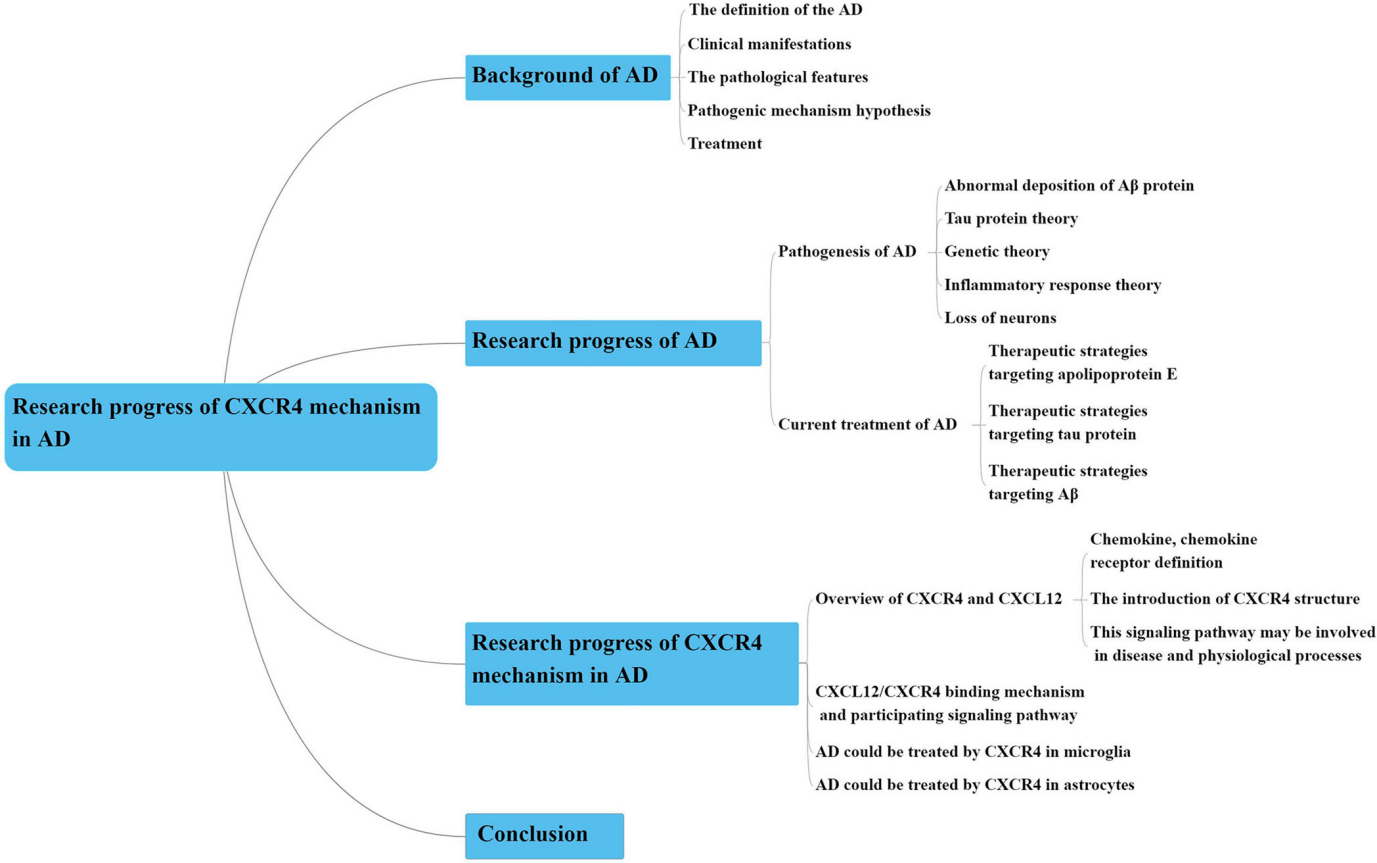
Mind map Text flow chart. AD, Alzheimer's disease; CXCR4, chemokine receptor type 4 [Color figure can be viewed at wileyonlinelibrary.com]

In this review, we have summarized the mechanism of AD and therapeutics of AD, with a focus on the structure of CXCR4 and ligand CXCL12 and the mechanism of CXCR4/CXCL12, which are involved in the occurrence and development of AD. The possible therapeutics of AD through microglia and astrocytes have also been described, aiming to provide a new method for the treatment of AD. The specific mechanism of the CXCL12/CXCR4 axis and its interaction with other signaling pathways need to be further elucidated. Although the current level of research has achieved some results, it is still difficult to specifically control the growth, differentiation, and apoptosis of cells for the molecular treatment of AD. The following problems remain to be solved: (1) the specific role of CXCR4 in the development of AD remains unclear; (2) How the CXCL12/CXCR4 axis regulates downstream molecules to affect AD needs to be further determined; and (3) how the CXCL12/CXCR4 axis interacts with other pathways to affect the occurrence and development of AD has not been clarified. Therefore, it is necessary to further study the involvement of this pathway in the occurrence and development of AD, so as to provide theoretical and experimental bases for the clinical treatment of AD (the full‐text flow chart is shown in Figure 4).

## CONFLICTS OF INTEREST

The authors declare no conflicts of interest.

## ETHICS STATEMENT

Not applicable.

## AUTHOR CONTRIBUTIONS

*Conceptualization*: Qiu‐Lin Wang was involved in the conceptualization of this study; Qiu‐Lin Wang, Chang‐Le Fang, and Lu‐Lu Xue were involved in writing, reviewing, and editing this manuscript; and Lu‐Lu Xue and Xue‐Yan Huang were involved in visualization, investigation, and revision of the draft of this manuscript.

## Data Availability

The data used to support the findings of this study are available from the corresponding author upon request.
